# Klinische Diagnostik, Differenzialdiagnostik, Pathogenese- und Stadienmodell der Arthrofibrose

**DOI:** 10.1007/s00113-022-01237-1

**Published:** 2022-09-15

**Authors:** Philipp Traut

**Affiliations:** Praxis für orthopädische Beratung und Begutachtung, Dörgen 31, 32549 Bad Oeynhausen, Deutschland

**Keywords:** Gelenkersatz, Knie, Kreuzband, Gelenksteifheit, Vernarbung, Joint replacement, Knee, Cruciate ligament, Stiffness, Scarring

## Abstract

Die Arthrofibrose (AF) gehört zu den häufigsten Komplikationen nach Verletzungen und operativen Eingriffen an Gelenken, v. a. nach Gelenk- und Kreuzbandersatz. Alle großen Gelenke können betroffen sein, am häufigsten ist es jedoch das Kniegelenk. Es kommt zur schmerzhaften Bewegungseinschränkung durch Vermehrung von fibrotischem Gewebe innerhalb und teilweise auch außerhalb des Gelenks. Der normale Heilungsprozess ist durch mechanische und emotionale Stressoren sowie starke Schmerzreize gestört. Die AF tritt zu 90 % schon wenige Tage nach der Verletzung oder Operation auf, sodass die Qualitätsstandards nicht erreicht werden können. Durch Physiotherapie und Rehabilitation kann oft keine wesentliche Verbesserung der Funktion erreicht werden, sodass die Aktivitäten des täglichen Lebens (ADL) stark eingeschränkt sind. Klinische Diagnostik, Differenzialdiagnostik sowie ein neues Pathogenese- und Stadienmodell der primären AF mit den daraus abgeleiteten therapeutischen Prinzipien werden vorgestellt.

## Abstract (en)

Arthrofibrosis (AF) is one of the most frequent complications of injuries and surgical interventions on joints, particularly after total knee replacement and anterior cruciate ligament reconstruction. Even though all joints can be affected, most cases involve the knee joint. Patients feel a painful impairment of the range of motion caused by fibrotic tissue formation within and sometimes also outside the joint. The normal healing process is disturbed by mechanical and emotional stressors and severe pain. In 90% of the cases AF occurs even within the first few days after the injury or surgery, so that the quality standards cannot be achieved. Physiotherapy and rehabilitation often do not result in a substantial amelioration of symptoms, so that the activities of daily living (ADL) are severely limited. The clinical diagnostics, differential diagnostics and a novel pathogenesis and stage model of the primary AF with the treatment principles derived from this are presented in this article.

Die Arthrofibrose (AF) gehört zu den häufigsten Komplikationen nach Verletzungen und operativen Eingriffen an Gelenken, v. a. nach Endoprothetik [4] und Kreuzbandersatz [2]. Alle großen Gelenke können betroffen sein, am häufigsten jedoch das Kniegelenk [8]**.** Schon wenige Tage nach dem Eingriff kommt es durch die Vermehrung von fibrotischem Gewebe innerhalb des Gelenks zu einer schmerzhaften Bewegungseinschränkung, sodass der bundeseinheitliche Qualitätsstandard bis zur Entlassung aus der Klinik nicht erreicht werden kann. Durch Physiotherapie und Rehabilitation kann oft keine wesentliche Verbesserung der Funktion erreicht werden, sodass später die Aktivitäten des täglichen Lebens (ADL) stark eingeschränkt sind. Narkosemobilisationen und Arthrolysen bei AF-Patienten werden in letzter Zeit zunehmend kritisch gesehen, da sie meist nur eine kurzfristige Besserung der Funktion bewirken. In der Literatur wird eine Rezidivrate von ca. 70 % angegeben [4, 5].

## Häufigkeit und Vorkommen

Das Auftreten der AF wird unterschiedlich hoch eingeschätzt. In der Literatur werden Zahlen von 1–13 % genannt [4]. In der Deutschen Gesellschaft für Endoprothetik (AE) ist eine Häufigkeit von 6–10 % nach primären Kniegelenkersatz (Totalendoprothese, TEP) allgemein anerkannt. Für die anderen Gelenke liegen keine Schätzungen vor. Die entsprechende Häufigkeit dürfte aber deutlich geringer sein, v. a. beim Hüftgelenkersatz. Diese Beobachtung korreliert auch mit der deutlich höheren Unzufriedenheitsrate von etwa 20 % in der Knieendoprothetik [3].

Die jährlichen Neuerkrankungen der AF können in Deutschland insgesamt auf etwa 50.000 geschätzt werden, allein 18.000 nach Implantationen von Knie-TEP. Frauen sind bei dieser Erkrankung deutlich häufiger betroffen als Männer, etwa im Verhältnis 3:2. Es sind zwei Häufigkeitsgipfel zu beobachten, zwischen 20 und 30 sowie 55 und 65 Jahren. Bei den jüngeren Patient*innen handelt es sich im eigenen Kollektiv meist um Sportverletzungen mit Kreuzbandersatz und bei der älteren Gruppe um degenerative Veränderungen nach Einsatz einer Endoprothese.

## Pathogenesemodelle

### Aktuelles Krankheitsmodell

Üblicherweise wird die AF als „Verklebung“ bezeichnet, und daraus werden auch die therapeutischen Maßnahmen abgeleitet. Kenntnisse über die biochemischen und biologischen Zusammenhänge dieser Heilungsstörung bestehen in der orthopädischen Praxis eher weniger. Es wird durch intensive Physiotherapie mit postisometrischen Dehnübungen und Motorschienen versucht, die nach einem operativen Eingriff eingetretene Bewegungseinschränkung mechanisch zu verbessern. Dies führt meist nicht zum erhofften Erfolg, sondern in vielen Fällen sogar zu einer Verschlechterung der Funktion mit starken Schmerzen trotz monatelanger Therapie. Dieses weitverbreitete „Verklebungsmodell“ sollte deshalb infrage gestellt werden.

**Figure 1:**
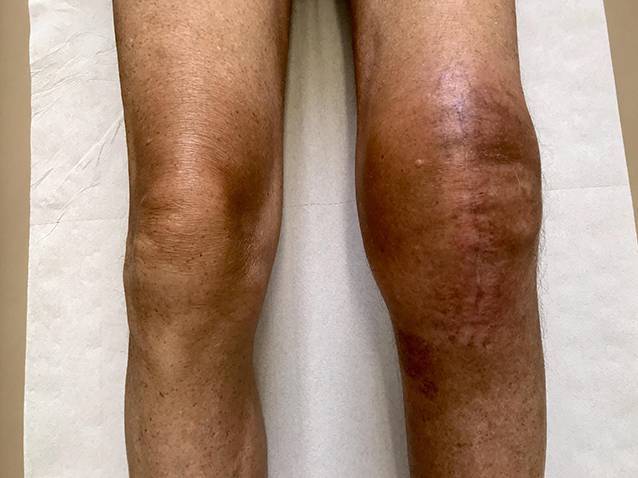

In Abb. 1 ist das Vollbild einer AF 6 Monate postoperativ nach einer linksseitigen Knie-TEP sowie frustraner physiotherapeutischer und chirurgischer Behandlung dargestellt. Es bestehen ein Streck- und Beugedefizit, eine Kapselschwellung, eine Überwärmung, ein „Schraubstockgefühl“, eine immobile Kniescheibe sowie ein starker Anlaufschmerz morgens und nach längerem Sitzen. Bei dieser „späten Diagnose“ ist mit einem längeren Heilungsverlauf zu rechnen. Nicht immer ist in diesen Fällen eine befriedigende Funktion des betroffenen Gelenks zu erreichen.

### Zelluläres, zytokinbasiertes Krankheitsmodell

Im Jahr 2015 ist ein neues Pathogenesemodell der AF veröffentlicht worden; dieses stützt sich auf Ergebnisse aus der Fibroseforschung [1, 6, 9, 10, 29]. Die gewonnenen Erkenntnisse können weitgehend auf die AF übertragen werden. Dieses Krankheitsmodell berücksichtigt die zellulären und biochemischen Vorgänge während des Heilungsprozesses [29]. Die Untersuchungen haben gezeigt, dass die Fibroblasten mechanisch sensibel sind und mechanischer Stress diese Zellen aktiviert und den natürlichen Zelltod (Apoptose) verhindert [10]**. **Dadurch kommt es zu einer starken Zell- und Bindegewebevermehrung. Dieses Gewebe wird bei der überwiegenden Mehrzahl der Betroffenen in sehr kurzer Zeit mit Schmerznerven versorgt. Dies erklärt die meist hohe Schmerzintensität bei der Mobilisation [14].

**Figure 2:**
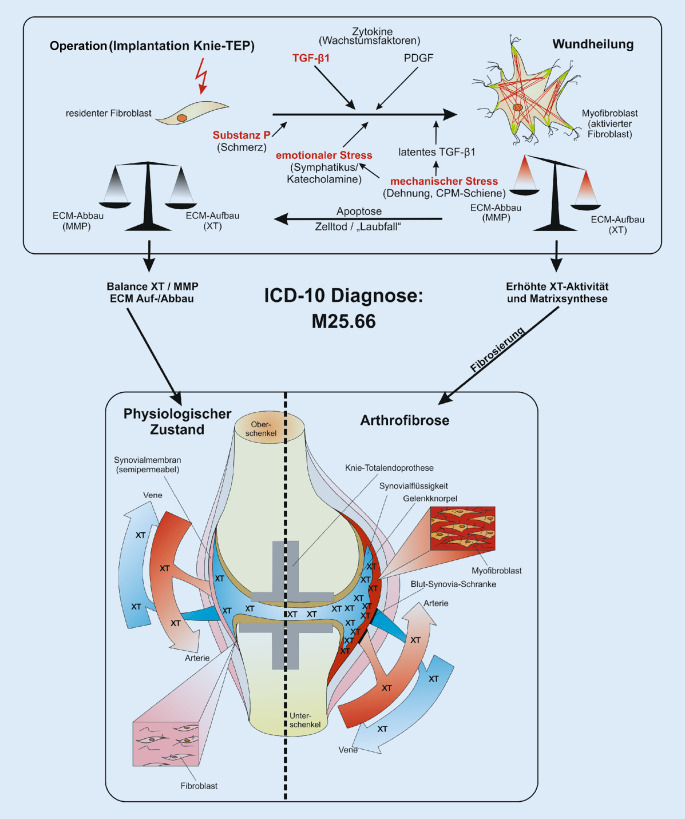

In Abb. 2 ist das zelluläre, zytokinbasierte Pathogenesemodell der AF nach Faust und Traut mit Darstellung der physiologischen und pathobiochemischen Vorgänge während der postoperativen Phase nach einem Kniegelenkersatz ersichtlich. Im Rahmen der physiologischen Wundheilung erfolgt eine Aktivierung residenter Fibroblasten zu Myofibroblasten. Hierbei spielen nicht nur Zytokine eine Rolle, sondern auch mechanischer und emotionaler Stress. Kommt es zur geregelten Apoptose der Myofibroblasten, liegen Auf- und Abbau der extrazellulären Matrix (ECM) sowie die Synthese der Xylosyltransferase (XT) im Gleichgewicht. Eine AF entsteht, wenn die Myofibroblasten persistieren sowie eine erhöhte ECM-Synthese und XT-Aktivität vorliegen [6, 24].

Neben dem mechanischen Stress konnte auch der emotionale Stress als Ursache für die AF erkannt werden. Stresshormone haben in der Zellkultur zu einer Vermehrung von kardialen Fibroblasten geführt [27]. Dies deckt sich mit einer amerikanisch-ungarischen Studie, in der Veränderungen des Stoffwechsels und des Apoptoseverhaltens der Fibroblasten bei Patienten mit einer Depression festgestellt wurden [7]. Bei der Herzfibrose (Tako-Tsubo-Kardiomyopathie) ist bekannt, dass allein emotionaler Stress diese schwere oft sogar tödliche Erkrankung auslösen kann [22]. Auch bei AF-Patienten ist häufig eine emotionale Belastungssituation, die vielfache Ursachen haben kann, nachweisbar. In seltenen Fällen kann die Fibrosierung des Gelenks mehrere Monate nach einem Gelenkersatz allein durch eine außerordentliche emotionale Belastung ausgelöst werden (Suizidversuch; *eigenes Patientengut*).

Neben mechanischem wurde auch emotionaler Stress und Schmerz als Ursache der AF identifiziert

Bei der schmerzhaften Dehnung des betroffenen Gelenks wird außerdem der Neurotransmitter „Substanz P“ freigesetzt, der die AF durch eine Aktivierung der Wachstumsfaktoren verstärkt [13, 17]. Aufgrund der Fibroseforschung können somit 3 Faktoren benannt werden, die zu einer möglichen Ausbildung der AF beitragen können. Auf dem Boden dieses Krankheitsmodells ist ein Therapiekonzept entwickelt worden, das die genannten Ursachen dieser Erkrankung berücksichtigt.

## Hypothetisches Stadienmodell der primären Arthrofibrose

**Figure 3:**
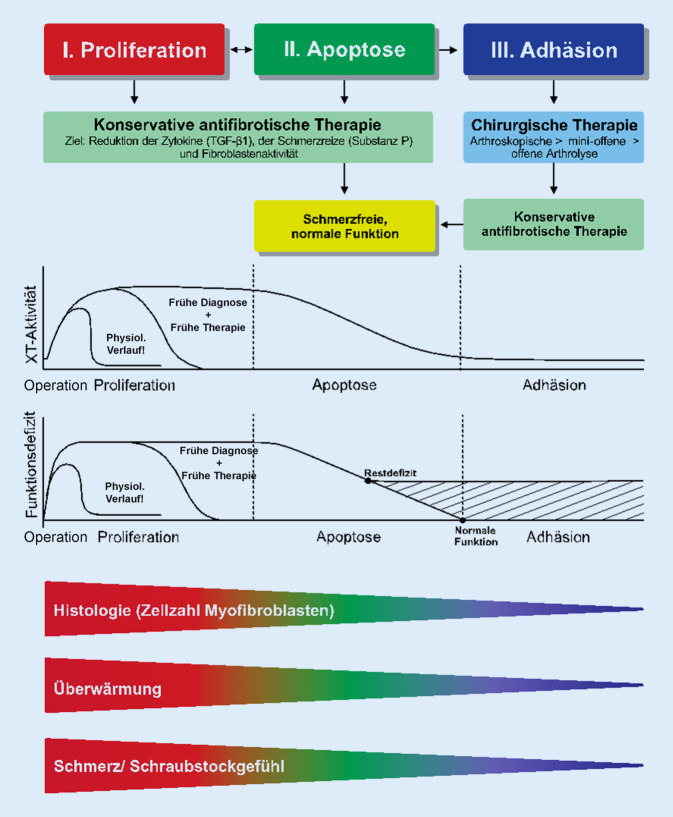

Ähnlich wie beim „complex regional pain syndrome“ (CRPS) ist ein Stadienverlauf der AF zu beobachten, der in dem hypothetischen Modell mit den klinischen, laborchemischen, histologischen und therapeutischen Besonderheiten beschrieben wird (Abb. 3). Es lassen sich ein Stadium der Proliferation (I), der Apoptose (II) und der Adhäsion (III) abgrenzen; diese erfordern unterschiedliche therapeutische Maßnahmen.

### Stadium I (Proliferation)

Im Stadium I kommt es durch die Zytokine „transforming growth factor beta 1“ (TGF-β1) und „platelet-derived growth factor“ (PDGF) zur Vermehrung der Fibroblasten und der ECM, die im physiologischen Verlauf in wenigen Tagen rückläufig ist. Bei Persistenz ist therapeutisch eine Reduktion der Zytokine und der Freisetzung der „Substanz P“ durch Verzicht auf schmerzhafte Dehnungsreize anzustreben, um die Apoptose der Myofibroblasten und den Abbau der fibrotischen ECM durch Matrixmetalloproteinasen (MMP) zu fördern. Die Aktivität der Myofibroblasten kann zusätzlich „off label“ medikamentös durch Gaben von Prednisolon und dem nichtselektiven β‑Blocker Propanolol gedämpft werden [28]**.**

In diesem Stadium sind chirurgische Maßnahmen, wie Arthrolysen und Narkosemobilisationen, mit einer hohen Rezidivrate belastet, da sie mit deutlichem mechanischen Stress und einer extremen Zytokinfreisetzung verbunden sind [4, 5]. Nach einer kurzfristigen postoperativen Besserung der Funktion des betroffenen Gelenks kommt es im weiteren Verlauf oft zur erneuten Verschlechterung.

### Stadium II (Apoptose)

Mithilfe einer konservativen antifibrotischen Therapie kann das Stadium II der Apoptose erreicht werden; in diesem Stadium sterben die Myofibroblasten ab und das extrazelluläre, fibrotische Gewebe wird durch MMP degradiert und abgebaut. Wenn das Stadium I nur wenige Monate angedauert hat, verbessert sich das Funktionsdefizit mit zunehmendem Abbau der ECM und Apoptose der Myofibroblasten. Dies zeigt sich klinisch am Rückgang der Überwärmung und der Schmerzen, histologisch vermutlich in der Verringerung der Zellzahl. Laborchemisch wird eine Reduktion der XT-Aktivität angenommen.

### Stadium III (Adhäsion)

Wenn das Proliferationsstadium durch ineffiziente physiotherapeutische und chirurgische Maßnahmen zeitlich über ein Jahr verlängert wird, kann aufgrund der lange andauernden fehlenden Entfaltung der Recessus eine Adhäsion der Synovialblätter resultieren. Dies bedingt ein Funktionsdefizit, obwohl sich die reparative Störung gebessert hat. Eine niedrige XT-Aktivität wird auch in diesem Stadium erwartet.

Im Stadium III der Adhäsion erscheint eine konservative antifibrotische Therapie nicht mehr zielführend, sondern eine arthroskopische Arthrolyse mit Lösen der synovialen Gleitflächen. Die Rezidivgefahr dürfte wegen fehlendem oder wenig aktivem fibrotischem Gewebe deutlich geringer sein als in den Stadien I und II. Jedem chirurgischen Eingriff sollte zur Rezidivprophylaxe eine konservative antifibrotische Nachbehandlung folgen. Intensive schmerzhafte Dehnübungen und repetitive zyklische Mobilisationen („continuous passive motion“, CPM) sollten unterbleiben [11, 15, 18–21, 24]**.**

## Vorsorge und Prävention

Um das Risiko dieser Erkrankung zu mindern, sollten nach Möglichkeit keine Patienten operiert werden, die sich in einer schwierigen emotionalen Situation befinden. Eine bestehende Depression sollte zuerst fachärztlich und psychotherapeutisch behandelt werden. Auch private und berufliche Belastungsfaktoren sollten vom Operateur angesprochen und, so weit wie möglich, vor dem chirurgischen Eingriff behoben werden. Negativ wirken sich des Weiteren postoperative Komplikationen und klinische Versorgungsdefizite aus. Ein fehlplatzierter Schmerzkatheter ohne genügende alternative Schmerzlinderung kann in den ersten postoperativen Tagen zur Ausschüttung der „Substanz P“ führen, durch die die AF mitverursacht werden kann [13, 17]**.**

## Primäre und sekundäre Arthrofibrose

Zwei Arten der AF, die differenziert betrachtet werden müssen, sind in der Literatur beschrieben [19]. Die primäre AF betrifft das ganze Gelenk und wird durch die oben aufgeführten Ursachen hervorgerufen. Es besteht die typische Symptomatik mit „Schraubstockgefühl”, mehr als 2 °C-Überwärmung, Patella-Immobilität und schmerzhafter „Range-of-motion“(ROM)-Einschränkung unter Extension/Flexion (E/F) 0‑0-90 ohne chirurgische Ursachen im Gegensatz zur sekundären AF (Tab. 1). Diese hat meist eine mechanische oder infektiöse Ursache. Durch Instabilität oder Fehlstellung der Endoprothese oder falsche Position des implantierten Kreuzbands wird ein permanenter mechanischer Reiz ausgelöst, der eine meist lokal begrenzte Ansammlung von fibrotischem Gewebe (Zyklops) bewirkt. Diese mechanische Ursache lässt sich nur operativ durch die Korrektur der zugrunde liegenden pathologischen Veränderung behandeln. Oft können beide AF-Arten gleichzeitig an dem betroffenen Gelenk beobachtet werden; diese sollten auch beide differenziert behandelt werden.

**Table 1:** 

Symptome	Primär	Sekundär
„Schraubstockgefühl“	+++	+
Überwärmung	+++	+
Patella-Immobilität	+++	–
ROM-Einschränkung	+++	+
Weitere pathologische Gelenkveränderungen	–	+++

*ROM* „range of motion“

## Primäre Arthrofibrose

### Symptome und aktueller Behandlungsverlauf

Bei der primären AF treten in über 90 % der Fälle schon wenige Tage nach der Operation typische Beschwerden und Befunde auf, die eine frühe Diagnose ermöglichen. Die Patienten klagen über ein „Schraubstockgefühl“ und starke Schmerzen bei der Mobilisation. Die typischen Befunde sind eine immobile Kniescheibe sowie ein Streck- und/oder Beugedefizit von weniger als 0‑0-90 Grad. Diese Krankheitszeichen werden oft von den Behandlern nicht wahrgenommen. Mit intensiven Dehnübungen wird versucht, den Qualitätsstandard von freier Streckung und mindestens 90-Grad-Beugung noch bis zur Entlassung aus der stationären Behandlung zu erreichen. Dieses Vorgehen ist für die Betroffenen in den meisten Fällen mit starken Schmerzen verbunden.

In der anschließenden Rehabilitation oder ambulanten Physiotherapie wird weiterversucht, das Gelenk mithilfe passiver Dehnübungen gemäß dem „Verklebungsmodell“ zu mobilisieren. Trotz intensiver Therapie kann oft keine wesentliche Besserung der Beweglichkeit und der Schmerzen erreicht werden. In vielen Fällen tritt sogar eine Verschlechterung ein. Die Patienten erhalten meist keine plausible Erklärung für diesen frustranen Verlauf. Es wird oft zu Geduld und stärkerem Üben in Eigenregie geraten. Bei Entlassung ist alternierendes Treppensteigen und Ergometerfahren oft noch nicht möglich.

Bei der erneuten Vorstellung beim Operateur wird oft zu einer Narkosemobilisation, operativen Entfernung des „Narbengewebes“ oder weiterer intensiver Physiotherapie geraten. Narkosemobilisationen und arthroskopische bzw. offene Arthrolysen bewirken meist nur kurze Zeit eine Besserung der Funktion. Auch Prothesenwechsel sind in der frühen Phase der Erkrankung mit einer hohen Rezidivrate über 70 % belastet [4]. Im weiteren Verlauf kommt es oft zur zunehmenden Einsteifung des betroffenen Gelenks, sodass viele Aktivitäten des täglichen Lebens nicht mehr möglich sind. Bei Patienten im Erwerbsleben und geforderter körperlicher Belastung ist die berufliche Existenz gefährdet.

### Neues alternatives Therapiekonzept

Durch die Behandlung der primären AF nach dem zellulären, zytokinbasierten Pathogenesemodell auf der Grundlage der Fibroseforschung, die erstmals 2018 in *Orthopädie & Rheuma* beschrieben wurde [24], kann dieser für Patient und Operateur unerfreuliche Verlauf in den meisten Fällen verhindert werden (Abb. 2). Je früher die Therapie beginnt, desto schneller und effektiver lässt sich diese reparative Störung konservativ bessern [21, 23–25]**.** Je später diese kausale antifibrotische Therapie beginnt, desto weiter schreitet die Erkrankung mit zunehmenden strukturellen Veränderungen fort. In dem späten Stadium III der AF sind meist operative Maßnahmen notwendig (Abb. 3).

Der frühe Beginn der kausalen antifibrotischen Therapie bessert die reparative Störung effektiv

Vorbild kann die Behandlung der „Herzfibrose“ sein, die bei früher Diagnose und mechanischer Entlastung des Herzens innerhalb von 6 bis 8 Wochen zur völligen Heilung dieser schwerwiegenden, oft auch tödlichen Erkrankung führt [22]**.**

### Diagnose

Die Diagnose kann mit ausreichender Sicherheit anhand von 2 typischen Beschwerdeangaben und 2 charakteristischen Befunden schon im Akuthaus klinisch gestellt werden, spätestens jedoch nach der Rehabilitation. Eine histologische Untersuchung empfiehlt sich in Zweifelsfällen [16]. Auch wenn bei einer falsch-positiven Diagnose keine AF vorliegen sollte, wirkt sich die antifibrotische Therapie nicht negativ auf den weiteren Verlauf aus. Eine falsch-negative Diagnose kann dagegen für die Betroffenen zum monate- oft jahrelangem Krankheitsverlauf führen. Der Rat zu weiterer Geduld sollte nur dann geäußert werden, wenn die Ursache des verzögerten Heilungsverlaufs erkannt und die notwendigen therapeutischen Schritte eingeleitet wurden. Die konservative Behandlung einer AF kann durchaus 6 bis 12 Monate in Anspruch nehmen.

- *Beschwerden*: *1.* „Schraubstockgefühl“, *2.* starke Schmerzen bei der Mobilisation,
- *Befunde: 3.* Beweglichkeit weniger 0‑0-90 Grad, *4.* immobile Kniescheibe.

Die laborchemische Untersuchung der „XT-Aktivität“ in Serum und Gelenkpunktat wird aktuell in einer multizentrischen Studie evaluiert und kann zurzeit noch nicht diagnostisch eingesetzt werden. Dieses Enzym spiegelt die Fibroblastenaktivität im Gelenkbinnenraum wider. Aktuell kann die XT schon als Biomarker in der Diagnostik der Sklerodermie und Leberfibrose genutzt werden [9]**.**

In späteren Stadien können weitere typische Beschwerden auftreten und Befunde nachgewiesen werden, die die Diagnose zusätzlich sichern können (Abb. 1)**.**

## Differenzialdiagnosen bei schmerzhaften Gelenkbeschwerden

Auch andere Erkrankungen können mit einer schmerzhaften Bewegungseinschränkung einhergehen und sollten im Behandlungsverlauf beachtet werden. Hierzu zählen:

- *postoperative Narbenneurome*,
- Lockerung der Endoprothese,
- Retropatellararthrose,
- Fibromyalgie
- *CRPS (M. Sudeck)*,
- Gelenkinstabilitäten oder zu straffe Bandführung,
- „overstuffing“ (falsche Prothesengröße),
- Fehlposition der Endoprothese oder des Transplantats,
- *„Low-grade“-Infektion (chronische geringfügige Infektion)*,
- somatoforme Schmerzstörung, dissoziative Störung.

Alle diese Erkrankungen zeichnen sich durch andere typische Beschwerden und Befunde aus, sodass sie gut von der primären AF unterschieden werden können (Tab. 2). Die 3 kursiv gesetzten Diagnosen (s. oben) werden im Einzeln behandelt, da sie, ähnlich wie die AF, im postoperativen Verlauf häufig nicht erkannt werden. Dies führt zu unnötigen Fehlbehandlungen.

**Table 2:** 

	Bewegungseinschränkung	Patella-Immobilität	„Schraubstockgefühl“	Anlaufschmerz	Belastungsschmerz	Erguss	Überwärmung
Primäre Arthrofibrose	+++	+++	+++	+++	–	+	++
Postoperative Narbenneurome	++	–	–	+	+	–	+
Lockerung der Endoprothese	+	–	–	+++	+++	++	++
Retropatellararthrose	+	++	+	++	+++	+++	++
Fibromyalgie	–	–	–	++	+	–	–
CRPS (M. Sudeck)	+++	+++	+++	+++	+++	–	+++
Gelenkinstabilität oder zu straffe Bandführung	++	–	–	+	++	++	++
Overstuffing (falsche Prothesengröße)	++	+	++	+	–	+	+
Fehlposition der Endoprothese oder des Transplantats	++	+	–	–	+	–	+
Low-grade-Infektion (LGI)	+	–	+	+	++	+++	+++
Somatoforme, dissoziative Störung	++	–	–	–	–	–	–

*CRPS* „complex regional pain syndrome“

Die größte Übereinstimmung besteht zwischen AF und CRPS, sodass die letztere Erkrankung häufig nicht erkannt wird. Wichtige Unterscheidungsmerkmale sind der Belastungsschmerz und die sehr starke Überwärmung von 3–4 °C, die bei der AF meist fehlen. Beim „kalten“ CRPS liegt sogar eine Unterkühlung von bis zu 3°C vor, die bei der AF nie beobachtet werden kann. Zusätzlich sollte die bildgebende Untersuchung als diagnostische Hilfe genutzt werden. Beim CRPS sind destruktive Veränderungen, wie eine Patella baja sowie gelenk- und prothesennahe Entkalkungen nachweisbar, auch wenn sich die klinischen Symptome schon teilweise gebessert haben. Es können mehrere pathologische Veränderungen gleichzeitig auftreten; diese müssen weiterdifferenziert werden, um jede spezifisch behandeln zu können. Bei einer Low-grade-Infektion können z. B. zusätzlich eine AF und ein CRPS vorliegen.

### Postoperative Narbenneurome des N. saphenus

Diese postoperative Komplikation entwickelt sich erst in der 3. bis 4. Woche und löst typische Berührungsschmerzen im lateralen Narbenbereich aus. Es werden Schmerzen wie „Nadelstiche“ angegeben. Durch die Anspannung der Haut beim Beugen wird ebenfalls dieser typische Schmerz ausgelöst, sodass es zu einer Beugehemmung kommt. Deshalb besteht eine Verwechslungsgefahr mit der AF. Die Behandlung mit Pregabalin und Lidocainpflaster (Versatis®) ist meist erfolgreich, sodass neurochirurgische Eingriffe nur selten erforderlich sind.

### Complex regional pain syndrome (M. Sudeck)

**Figure 4:**
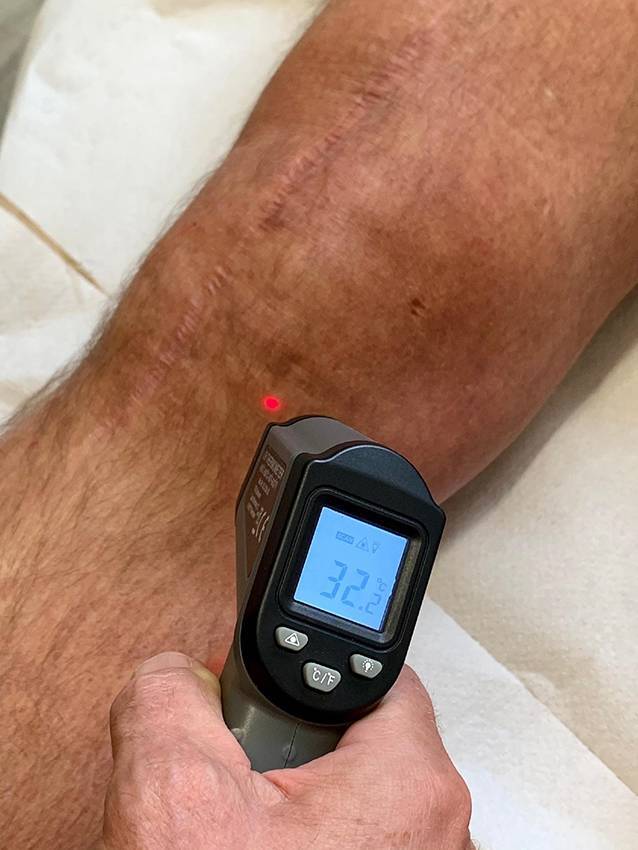

Arthrofibrose und CRPS stehen in einer besonderen Beziehung mit fließenden Übergängen zueinander. Aus einer AF kann sich bei weiterer Traumatisierung durch schmerzhafte Physiotherapie, Narkosemobilisationen oder operative Eingriffe ein CRPS entwickeln, was in der Literatur bei 15 % der Fälle beschrieben wird [20]. Jedes CRPS ist neben den typischen Beschwerden und Symptomen, die in den „Budapest-Kriterien“ zusammengefasst sind, häufig mit einer schmerzhaften Bewegungseinschränkung belastet. Ruheschmerz, Hautberührungsempfindlichkeit, Muskelansteuerungsprobleme, trophische Störungen des gesamten Beins mit großen Temperaturdifferenzen über 3–4 °C zur Gegenseite und destruktive Veränderungen, wie Patella baja, sichern die Diagnose (Abb. 4). Jeder weitere operative Eingriff oder eine Narkosemobilisation kann zu einer dramatischen Verschlechterung führen. Eine schmerztherapeutische Behandlung oder stationäre Rehabilitation in einer spezialisierten Einrichtung ist fast immer indiziert.

### „Low-grade“-Infektion (chronische geringfügige Infektion)

Bei 1–2 % der Gelenkimplantate entwickelt sich diese schwerwiegende Komplikation [12]. Wird die Diagnose innerhalb der ersten 4 bis 6 postoperativen Wochen gestellt, ist ein Prothesenwechsel meistens vermeidbar. Bei deutlichem Gelenkerguss sollte direkt nach „Frösteln“, kurzfristigem Fieber und nachfolgendem Schwitzen gefragt werden (B-Symptomatik), da die Patienten diese Symptomatik nicht mit ihrem neuen Gelenk in Verbindung bringen. Eine sofortige Diagnostik mit Keimnachweis kann die Prothese „retten“. Um „falsch-negative“ Ergebnisse zu vermeiden, sollte die Gelenkpunktion in zeitlicher Nähe zum Fieberanstieg durchgeführt werden, da nur in diesem Zeitraum vitale Keime in der Synovialflüssigkeit vorhanden sind.

Bei später Diagnose ist meist ein Prothesenwechsel notwendig [12]. Liegt gleichzeitig ein CRPS vor, sollte die Operationsindikation wegen der möglichen schweren postoperativen Komplikationen streng gestellt werden. Eine palliative Langzeitantibiotikabehandlung ist in Erwägung zu ziehen [12]**.**

## Therapie

### Physio- und medikamentöse Therapie

Wenn die AF früh diagnostiziert wird, besteht die Chance der völligen Heilung. Bei späterer Diagnose kann noch eine deutliche Besserung durch eine konservative antifibrotische Therapie erzielt werden. Ziel der Therapie ist die Gesundung des betroffenen Gelenks. Es darf nicht versucht werden, ein krankes Gelenk beweglich zu machen, weil das intraartikuläre fibrotische Gewebe, das die Bewegung hemmt, immer wieder verletzt wird, mit nachfolgender nichtendender Reparation. Die Warnfunktion des Schmerzes sollte beachtet werden [26]. Nach Abbau des pathologischen Gewebes resultiert die physiologische Beweglichkeit des betroffenen Gelenks von selbst.

Der Abbau des pathologischen Gewebes resultiert in der physiologischen Beweglichkeit des Gelenks

Folgende therapeutische Maßnahmen sind zu empfehlen:

1. Aufklärung des Patienten über die Ursachen und die therapeutischen Möglichkeiten,
2. sofortiger Stopp der passiven schmerzhaften Mobilisation des Gelenks [11, 20, 21],
3. Bewegung nur im schmerzfreien Bereich erlaubt,
4. Förderung der Mikrozirkulation und Entsorgung der Zytokine (Wachstumsfaktoren) durch Lymphdrainagen, ZRT(zellbiologische Regulationstherapie)®-Matrix-Therapie, Therapie mithilfe pulsierender elektromagnetischer Felder (PEMF), Akupunktur,
5. Balancierung des vegetativen Systems durch Osteopathie, Fußreflexzonentherapie, Bindegewebsmassagen, evtl. psychologische Begleitung,
6. Erlernen von Entspannungstechniken (autogenes Training, Achtsamkeitstraining, progressive Muskelentspannung, Yoga u. a.),
7. medikamentöse Therapie mit niedrigdosiertem Prednisolon und dem nichtselektiven β‑Blocker Propanolol bei fehlenden Kontraindikationen [28]

### Ernährung, Bewegung und Entspannung in Eigenregie

Wichtig ist, dass die Patienten alles vermeiden, was zur Freisetzung der Wachstumsfaktoren und damit zur Aktivierung der Fibroblasten führt. Es dürfen nur die Bewegungen durchgeführt werden, die keine Schmerzen verursachen. Treppensteigen im Wechselschritt ist erst bei einer Beugung von etwa 100 Grad möglich und erlaubt, vorher nur im Beistellschritt. Auch Fahrrad- oder Ergometerfahren ist erst bei über 90-Grad-Beugung oder mit Kurbelverkürzung zur Kreislaufaktivierung therapeutisch sinnvoll. Längere Gehstrecken sind nur erlaubt, wenn das „Schraubstockgefühl“ danach nicht verstärkt auftritt. Aktuelles Prinzip: *Weniger ist mehr! *Auch ein Wellness-Urlaub ist zur Balancierung des vegetativen Systems sinnvoll.

### Medikamentöse Therapie

Für die AF steht aktuell nur eine Off-label-Therapie zur Verfügung; diese muss mit den Patienten besprochen werden. Es sind aber Medikamente (Prednisolon und Propanolol) verfügbar, die seit vielen Jahren in ihren Wirkungen und Nebenwirkungen bekannt sind. Bei Beachtung der Kontraindikationen können sie zusätzlich eingesetzt werden, v. a. bei starken Schmerzen in den Stadien I und II [28]**.** Sie haben allerdings nur eine symptomatische Wirkung, ohne direkte Förderung der Apoptose, sodass bei Gegenanzeigen auf ihren Einsatz verzichtet werden kann, ohne den Erfolg der Therapie zu gefährden. Eine fachlich richtige Physiotherapie und geeignete Verhaltensmaßnahmen der Betroffenen haben einen größeren Einfluss auf den Heilungserfolg.

### Nachsorge

Wenn die Behandlung erst im späten Stadium II oder III einsetzt, verbleibt oft eine Empfindlichkeit gegenüber mechanischem und/oder emotionalem Stress. Einige Patienten berichten, dass ihr Kniegelenk ein „Spiegel ihrer Seele“ sei und sich bei emotionaler Belastung verschlechtere. Für diese Patientengruppe ist es von großer Wichtigkeit, Techniken zur Reduktion von innerer Anspannung zu erlernen und regelmäßig einzusetzen. Es gibt auch viele Patienten, bei denen bei bestehender AF das kontralaterale Gelenk operiert werden musste. Bei den meisten vom Autor des vorliegenden Beitrags behandelten Patienten ist an dem anderen Gelenk keine AF aufgetreten, sodass wesentliche genetische Faktoren bei dieser Erkrankung eher nicht vorliegen. Vor einem operativen Gelenkeingriff sollten allerdings eine emotionale Ausgeglichenheit und ein ungetrübtes Vertrauensverhältnis zum Operateur bestehen.

## Fazit für die Praxis

- Die Arthrofibrose (AF) ist eine Erkrankung, die ärztlich und physiotherapeutisch gut behandelt werden kann. Die Behandler sollten den Mut haben, die Diagnose frühzeitig zu stellen und eine antifibrotische Therapie einzuleiten. Der Rat zu mehr Geduld sollte den betroffenen Patient*innen nur dann gegeben werden, wenn die Ursache der Funktionsstörung diagnostisch klar definiert wurde.
- Behandlungsziel bei diagnostizierter AF sollte die Gesundung durch Abbau des fibrotischen Gewebes im betroffenen Gelenk sein. Dann verringern sich die Schmerzen und das Schraubstockgefühl in relativ kurzer Zeit, und die Beweglichkeit verbessert sich. Die bisherige „mechanisch“ orientierte Behandlung nach dem „Verklebungsmodell“ mit passivem schmerzhaftem Dehnen sollte der Vergangenheit angehören.
- Da die betroffenen Patienten zuerst und am intensivsten durch Physiotherapeuten betreut und behandelt werden, können diese in besonderem Maß zum Therapieerfolg beitragen. Operateure und die weiteren ärztlichen Nachbehandler sollten die Therapeuten diagnostisch und beratend unterstützen.
